# The Study for Synchronization between Two Coupled FitzHugh-Nagumo Neurons Based on the Laplace Transform and the Adomian Decomposition Method

**DOI:** 10.1155/2021/6657835

**Published:** 2021-04-23

**Authors:** Bin Zhen, Zigen Song

**Affiliations:** ^1^School of Environment and Architecture, University of Shanghai for Science and Technology, Shanghai 200093, China; ^2^College of Information Technology, Shanghai Ocean University, Shanghai 201306, China

## Abstract

The synchronization between two coupled FitzHugh-Nagumo (FHN) neurons with or without external current is studied by using the Laplace transform and the Adomian decomposition method. Different from other researches, the synchronization error system is expressed as sets of Volterra integral equations based on the convolution theorem in the Laplace transform. Then, it is easy to analytically obtain the conditions that synchronization errors disappear based on the successive approximation method in integral equation theorem, the correctness of which is verified by numerical simulations. Furthermore, the synchronous dynamics of the two coupled FHN neurons also can be written in the form of Volterra integral equations, which is more convenient to analytically solve by using the Adomian decomposition method. It is found that the occurrence of synchronization between the two FHN neurons only depends on the coupling strength and is irrelevant to the external current. Only synchronous rest state in the two FHN neurons without external current can be achieved, while synchronous spikes appear if the external current is not zero.

## 1. Introduction

The neurons are considered as basic units to form neural systems, which have received much interest because of the great significance and applications in neural science, brain science, medical technology, and so on. To simplify matters, the Hodgkin–Huxley (HH) equation [1–3] is usually used to investigate the dynamics of neural networks. Although the HH equation and its modified versions are simplification of dynamics of a realistic neuron, it is still complex in some problems. A simplified model of the HH equation called the FitzHugh-Nagumo (FHN) model [4] is convenient in the investigation of neural behavior by using nonlinear dynamical theory [5, 6]. The FHN model has cubic nonlinearity, which can show excitability of a neuron. The qualitative nature of nerve impulse propagation and neural activity can exhibit in several coupled FHN models, such as separatix loops and bifurcation of equilibria and limit cycles [7, 8].

Synchronization in neural systems is a crucial phenomenon. In many regions of the brain, synchronization is considered to be related to cognition as well as the correlate of behavior [3, 9]. Phase synchronization [10] may play a very important role in the brain's ability to store, process, and communicate information. Synchronization caused by parameter mismatch [11] is a more general case in the real world. Synchronization and corresponding dynamics of coupled neurons have been studied in [12, 13] to understand information processing in the brain. The effects of Gaussian white noise on synchronization were researched in [14].

In the past decade, many criteria have been given to judge the occurrence of synchronization in dynamical systems. The Lyapunov function technology is one of the earliest methods to establish the synchronization conditions in coupled oscillators. The famous master stability function (MSF) method is very effective to analyze the local stability of the synchronization manifold [15]. Chen [16] proposed the matrix measure approach (MMA) to analytically derive sufficient conditions for synchronization in time-varying networks. Lu and Chen [17] established the synchronization conditions for linearly coupled ordinary differential systems by considering the distance between trajectories and the synchronization manifold. The key to the Lyapunov function method is to find an appropriate Lyapunov function. Usually, the synchronization conditions by using such technology are sufficient and highly conserved. The study in [18] showed that the method may be invalid for systems with multiple components of different types. In the MSF method and the MMA, the conditional Lyapunov exponents have to be calculated numerically. For the new approach given by Lu and Chen, some information of the synchronous dynamics needs to be known previously. However, the trajectories in the synchronization manifold are unknown before solving the coupled systems. According to the statements above, the analytical criterion for synchronization occurrence is still a question worthy to further discuss.

In this paper, we use the Laplace transform to investigate the synchronization conditions for two coupled FHN neurons. Different from other studies, the synchronization error system derived from the original coupled neural system is converted into sets of Volterra integral equations based on the convolution theorem in Laplace transform. It should be noted that the technique that converts an ordinary differential equation into sets of Volterra integral equations has been successfully applied to other nonlinear systems. From References [19, 20], it was found that the form of the Volterra integral equations is clearer to show the influence of nonlinear parts in the governing equation of a beam under a moving load. According to the successive approximation method [21] in the integral equation theory, it is convenient to obtain the conditions that synchronization errors disappear. Furthermore, the governing equation describing the synchronous dynamics of the two FHN neurons also can be written in the form of the Volterra integral equation, which is easy to analytically solve by using the Adomian decomposition method [22]. Numerical simulations are carried out to demonstrate the correctness of the analytical approach proposed in this paper. It is analytically verified that two coupled FHN neurons without external current can only exhibit synchronization rest regardless of the coupling strength. When the external current is not zero, synchronization spikes between the two FHN neurons occur, and the synchronization conditions for coupling strength remain the same as that for the case of no external current.

The rest of the paper is organized as follows. In Section 2, the synchronization conditions for two coupled FHN neurons are discussed by using the Laplace transform. In Section 3, the synchronous dynamics of the coupled FHN neurons is analytically solved by using the Adomian decomposition method. In Section 4, numerical simulations are carried out to verify the effectiveness of the analytical results. Conclusions are given in Section 5.

## 2. Synchronization Conditions for Two Coupled FHN Neurons

A single FHN neuron is given in the following form:

(1) $$\begin{array}{c} {\dot{u}}_{1}=-u_{1}\left(u_{1}-1\right)\left(u_{1}-a\right)-u_{2}+I\sin\left(\omega t\right), \end{array}$$

(2) $$\begin{array}{c} {\dot{u}}_{2}=b\left(u_{1}-\gamma u_{2}\right), \end{array}$$

where *u*_1_ is the potential difference and *u*_2_ is a recovery current. *I* and *ω* are the magnitude and frequency of the external current, respectively. 0 < *a* < 0.5, *b*, *γ* > 0, (1 − *a* + *a*^2^)/3 > *bγ*, and (1 − *a* + *a*^2^)/3 < 1/*γ*. (0, 0) is the unique equilibrium point needed to be considered in system (1). Two FHN neurons are coupled as follows:

(3) $$\begin{array}{c} {\dot{u}}_{1}=-u_{1}\left(u_{1}-1\right)\left(u_{1}-a\right)-u_{2}+c\left(u_{1}-u_{3}\right)+I\sin\left(\omega t\right), \end{array}$$

(4) $$\begin{array}{c} {\dot{u}}_{2}=b\left(u_{1}-\gamma u_{2}\right), \end{array}$$

(5) $$\begin{array}{c} {\dot{u}}_{3}=-u_{3}\left(u_{3}-1\right)\left(u_{3}-a\right)-u_{4}+c\left(u_{3}-u_{1}\right)+I\sin\left(\omega t\right), \end{array}$$

(6) $$\begin{array}{c} {\dot{u}}_{4}=b\left(u_{3}-\gamma u_{4}\right), \end{array}$$

where *c* is the coupling strength. The two FHN neurons are said to be synchronous if $\underset{t\to +\infty }{\lim}u_{1}=u_{3}$ and $\underset{t\to +\infty }{\lim}u_{3}=u_{4}$. By letting

(7) $$\begin{array}{c} e_{1}=\frac{u_{1}-u_{3}}{2},\\ e_{2}=\frac{u_{2}-u_{4}}{2},\\ e_{3}=\frac{u_{1}+u_{3}}{2},\\ e_{4}=\frac{u_{2}+u_{4}}{2}, \end{array}$$

system (3) can be written as

(8) $$\begin{array}{c} {\overset{\cdot }{e}}_{1}=-e_{1}^{3}+\left[-3e_{3}^{2}+2\left(a+1\right)e_{3}-a+2c\right]e_{1}-e_{2}, \end{array}$$

(9) $$\begin{array}{c} {\dot{\text{e}}}_{2}=b\left(e_{1}-\gamma e_{2}\right), \end{array}$$

(10) $$\begin{array}{c} {\dot{e}}_{3}=-e_{3}^{3}+\left(a+1\right)e_{3}^{2}-\left(3e_{1}^{2}+a\right)e_{3}+\left(a+1\right)e_{1}^{2}-e_{4}+I\sin\left(\omega t\right), \end{array}$$

(11) $$\begin{array}{c} {\dot{e}}_{4}=b\left(e_{3}-\gamma e_{4}\right). \end{array}$$

Then, the synchronization conditions of system (3) become $\underset{t\to +\infty }{\lim}e_{1,2}=0$. In the following, we use the Laplace transform method to obtain the condition that *e*_*i*_ → 0, *i* = 1, 2. The Laplace transform used in this paper is defined as follows:

(12) $$\begin{array}{c} {\hat{e}}_{i}\left(s\right)=L\left[e_{i}\right]=\int_{0}^{+\infty }e_{i}\left(t\right)e^{-st}dt,\\ e_{i}\left(t\right)=L^{-1}\left[{\hat{e}}_{i}\right]=\frac{1}{2\pi i}\int_{\sigma -i\infty }^{\sigma +i\infty }{\hat{e}}_{i}\left(s\right)e^{st}ds,\;i=1,2,3,4. \end{array}$$

Taking the Laplace transforms of both sides of Equation (9) and arranging them yield

(13) $$\begin{array}{c} \left(s+a-2c\right){\hat{e}}_{1}+{\hat{e}}_{2}=e_{10}+{\hat{F}}_{1}, \end{array}$$

(14) $$\begin{array}{c} \left(s+b\gamma \right){\hat{e}}_{2}+e_{20}=+b{\hat{e}}_{1}, \end{array}$$

(15) $$\begin{array}{c} \left(s+a\right){\hat{e}}_{3}+{\hat{e}}_{4}=e_{30}+\frac{\omega I}{s^{2}+\omega^{2}}+{\hat{F}}_{2}, \end{array}$$

(16) $$\begin{array}{c} \left(s+b\gamma \right){\hat{e}}_{4}=e_{40}+b{\hat{e}}_{3}, \end{array}$$

where *e*_*i*0_, *i* = 1, 2, 3, 4, are given initial values of system (9), and

(17) $$\begin{array}{c} {\hat{F}}_{1}=\int_{0}^{+\infty }\left[-e_{1}^{3}-3e_{1}e_{3}^{2}+2\left(a+1\right)e_{1}e_{3}\right]e^{-st}dt\equiv \int_{0}^{+\infty }\left[F_{1}\left(t\right)\right]e^{-st}dt,\\ {\hat{F}}_{2}=\int_{0}^{+\infty }\left[-e_{3}^{3}+\left(a+1\right)e_{3}^{2}-3e_{1}^{2}e_{3}+\left(a+1\right)e_{1}^{2}\right]e^{-st}dt\equiv \int_{0}^{+\infty }\left[F_{2}\left(t\right)\right]e^{-st}dt. \end{array}$$

Substituting the second and fourth equations into the first and third equations in system (14), respectively, produces

(18) $$\begin{array}{c} {\hat{e}}_{1}=\frac{\left(s+\alpha_{1}\right)e_{10}-e_{20}}{s^{2}+a_{2}s+a_{3}}+\frac{\left(s+\alpha_{1}\right){\hat{F}}_{1}}{s^{2}+a_{2}s+a_{3}}, \end{array}$$

(19) $$\begin{array}{c} {\hat{e}}_{3}=\frac{\left(s+\alpha_{1}\right)e_{30}-e_{40}}{s^{2}+a_{4}s+a_{5}}+\frac{\left(s+\alpha_{1}\right)\left(\omega I/\left(s^{2}+\omega^{2}\right)\right)}{s^{2}+a_{4}s+a_{5}}+\frac{\left(s+a_{1}\right){\hat{F}}_{2}}{s^{2}+a_{4}s+a_{5}}, \end{array}$$

where *α*_1_ = *bγ*, *α*_2_ = *a* + *bγ* − 2*c*, *α*_3_ = *b*[(*a* − 2*c*)*γ* + 1], *α*_4_ = *a* + *bγ*, and *α*_5_ = *b*(*aγ* + 1). Since there exist the simply linear relations between ${\hat{e}}_{2,4}$ and ${\hat{e}}_{1,3}$, respectively, only the dynamical behaviors of ${\hat{e}}_{1,3}$ need to be considered. Let *Φ*_*i*_(*t*), *i* = 1, 2, 3, 4, denote the following inverse Laplace transforms, respectively,

(20) $$\begin{array}{c} \Phi_{1}\left(t\right)=L^{-1}\left[\frac{s+a_{1}}{s^{2}+a_{2}s+a_{3}}\right],\\ \Phi_{2}\left(t\right)=L^{-1}\left[\frac{1}{s^{2}+a_{2}s+a_{3}}\right],\\ \Phi_{3}\left(t\right)=L^{-1}\left[\frac{s+a_{1}}{s^{2}+a_{4}s+a_{5}}\right],\\ \Phi_{4}\left(t\right)=L^{-1}\left[\frac{1}{s^{2}+a_{4}s+a_{5}}\right]. \end{array}$$

Then, *Φ*_*i*_(*t*), *i* = 1, 2, 3, 4, can be given by

(21) $$\begin{array}{c} \left\{\begin{array}{c} \Phi_{1}\left(t\right)=\left\{\begin{array}{cc} e^{-\alpha_{2}t/2}\left[\cosh\left(\beta_{1}t\right)+\frac{\beta_{2}}{2\beta_{1}}\sinh\left(\beta_{1}t\right)\right], & \beta_{1}\neq 0,\\ e^{-\alpha_{2}t/2}\left(1+2\beta_{2}t\right), & \beta_{1}=0, \end{array}\right.\\ \Phi_{2}\left(t\right)=\left\{\begin{array}{cc} \frac{e^{-\alpha_{2}t/2}}{\beta_{3}}\sinh\left(\beta_{1}t\right), & \beta_{1}\neq 0,\\ {te}^{-\alpha_{2}t/2}, & \beta_{1}=0, \end{array}\right. \end{array}\right. \end{array}$$

(22) $$\begin{array}{c} \left\{\begin{array}{c} \Phi_{3}\left(t\right)=\left\{\begin{array}{cc} e^{-\alpha_{4}t/2}\left[\cosh\left(\beta_{3}t\right)+\frac{\beta_{4}}{2\beta_{3}}\sinh\left(\beta_{3}t\right)\right], & \beta_{3}\neq 0,\\ e^{-\alpha_{4}t/2}\left(1+2\beta_{4}t\right), & \beta_{3}=0, \end{array}\right.\\ \Phi_{4}\left(t\right)=\left\{\begin{array}{cc} \frac{e^{-\alpha_{4}t/2}}{\beta_{3}}\sinh\left(\beta_{3}t\right), & \beta_{3}\neq 0,\\ {te}^{-\alpha_{4}t/2}, & \beta_{3}=0, \end{array}\right. \end{array}\right. \end{array}$$

where $\beta_{1}=\sqrt{\alpha_{2}^{2}-4_{a3}}/2,\beta_{2}=2a_{1}-a_{2},\beta_{3}=\sqrt{\alpha_{4}^{2}-4_{a5}}/2,\beta_{4}=2a_{1}-a_{4}$. From the convolution theorem, taking the inverse Laplace transform on both sides of equations in Equation (18) yields

(23) $$\begin{array}{c} e_{1}=e_{10}\Phi_{1}-e_{20}\Phi_{2}+\int_{0}^{t}\Phi_{1}\left(t-\tau \right)F_{1}\left(\tau \right)d\tau , \end{array}$$

(24) $$\begin{array}{c} e_{3}=e_{30}\Phi_{3}-e_{40}\Phi_{4}+I\int_{0}^{t}\Phi_{3}\left(t-\tau \right)\sin\left(\omega \tau \right)d\tau +\int_{0}^{t}\Phi_{3}\;\left(t-\tau \right)F_{2}\left(\tau \right)d\tau , \end{array}$$

where *F*_1,2_ is defined in Equation (14). From Equations (21) and (22), if −(*a*_2_/2) ± |Re{*β*_1_}| < 0, Re{*β*_1_} represents the real part of *β*_1_, *Φ*_1,2_ decay to zero quickly. In this case, the first equation in Equation (23) is simplified as

(25) $$\begin{array}{c} e_{1}\left(t\right)=\int_{o}^{t}\Phi 1\left(t-\tau \right)F_{1}\left(\tau \right)d\tau =\int_{0}^{t}\Phi_{1}\left(t-\tau \right)\left[-e_{1}{\left(\tau \right)}^{2}-3e_{3}{\left(\tau \right)}^{2}+2\left(a+1\right)e_{3}\left(\tau \right)\right]e_{1}\left(\tau \right)d\tau . \end{array}$$

From the successive approximation method introduced in [21, 23], the recurrence relation

(26) $$\begin{array}{c} e_{1n+1}\left(t\right)=\int_{0}^{t}\Phi_{1}\left(t-\tau \right)\left[-e_{1n}{\left(\tau \right)}^{2}-3e_{3}{\left(\tau \right)}^{2}+2\left(a+1\right)e_{3}\left(\tau \right)\right]e_{1n}\left(\tau \right)d\tau ,n\geq 0 \end{array}$$

can be used to get the solution of Equation (25). Starting with an initial guess *e*_10_ = 0, it is clear that *e*_1_ = 0 is the solution of Equation (25) for any *e*_3_.If −*a*_2_/2 ± |Re{*β*_1_}| ≥ 0, *Φ*_1,2_ cannot approach to zero, then it means that *e*_1_ does not decay to zero. Thus, −*a*_2_/2 ± |Re{*β*_1_}| < 0 is a necessary and sufficient condition for *e*_1_ → 0 in system (9). It is easy to check that

(27) sign−α22+Reβ1=+,a2<0,sign−a3,a2>0&a22−4a3≥0,−,a2>0&a22−4a3<0,

(28) sign−α22−Reβ1=signa3,a2<0&a22−4a3≥0,+,a2<0&a22−4a3<0,−,a2>0.

Clearly, *α*_2_ = *a* + *bγ* − 2*c* > 0 must be satisfied to guarantee −*a*_2_/2 ± |Re{*β*_1_}| < 0. Under such condition, *α*_3_ = *b*[(*a* − 2*c*)*γ* + 1] > *b*(1 − *bγ*^2^) > 0 holds for the parameter ranges given in Equation (1). Therefore, *α*_2_ > 0(*c* < ((*a* + *bγ*)/2)) is the necessary and sufficient condition for *e*_1_ → 0 in system (9). Furthermore, if *α*_2_ > 0 and *a*_2_^2^ − 4*a*_3_ < 0, *e*_1_ approaches to 0 with oscillatory when *t* → +∞. If *α*_2_ > 0 and *a*_2_^2^ − 4*a*_3_ ≥ 0, *e*_1_ will lose its oscillatory behavior when itconverges to zero.

**Lemma 1 .**

For two coupled FHN neurons in system (1) with or without external current, synchronization occurs when and only when *c* < (*a* + *bγ*)/2.

**Remark 1 .**

Only when *Φ*_1,2_ defined in Equation (21) decay to 0, *e*_1_ → 0 for *t* → +∞. To guarantee *Φ*_1,2_ approach to 0, it is necessary and sufficient that the denominator in the first equation in Equation (18)

(29) $$\begin{array}{c} s^{2}+\alpha_{2s}+\alpha_{3} \end{array}$$

has two roots with negative real parts. According to the Routh-Hurwitz criterion, *α*_2,3_ > 0 are necessary and sufficient to guarantee that all roots of Equation (29) have negative real parts. Moreover, if polynomial (29) has two negative real roots, *e*_1_(*t*) is nonoscillatory.

## 3. Synchronous Dynamics of the Coupled FHN Neurons

In this section, we discuss the synchronous dynamics of system (3) with *I* = 0 and *I* ≠ 0, respectively.

### 3.1. *I* = 0 in System (3)

In this case, from the second equation in Equation (23), the governing equation of synchronous dynamics is given by

(30) $$\begin{array}{c} e_{3}=e_{30}\Phi_{3}-e_{40}\Phi_{4}+\int_{0}^{t}\Phi_{3}\left(t-\tau \right)F_{2}\left(\tau \right)d\tau =e_{30}\Phi_{3}-e_{40}\Phi_{4}+\int_{0}^{t}\Phi_{3}\left(t-\tau \right)\left[-e_{3}\left(\tau \right)+a+1\right]e_{3}{\left(\tau \right)}^{2}d\tau . \end{array}$$

Since *α*_4_ = (*a* + *bγ*) > 0 and −*a*_4_/2 ± |Re{*β*_3_}| < 0 hold according to the parameter ranges given in system (1), *Φ*_3,4_ defined in Equation (22) decay to zero very quickly when *t* → +∞. Using the successive approximation method, similar as that for Equation (25), the solution of Equation (30) is *e*_3_ = 0. Thus, if there is no external current in the coupled system, two FHN neurons only can achieve the synchronization of the resting state.

### 3.2. *I* ≠ 0 in System (3)

Under such condition, by getting rid of the terms including *e*^−*α*4*t*^ in the second equation in Equation (23), the governing equation of synchronous dynamics can be simplified as

(31) $$\begin{array}{c} e_{3}=\phi_{0}e^{i\omega t}+{\bar{\phi }}_{0}e^{-i\omega t}+\int_{0}^{t}\Phi_{3}\left(t-\tau \right)F_{2}\left(\tau \right)d\tau =\phi_{0}e^{i\omega t}+{\bar{\phi }}_{0}e^{-i\omega t}+\int_{0}^{t}\Phi_{3}\left(t-\tau \right)\left[-e_{3}\left(\tau \right)+a+1\right]e_{3}{\left(\tau \right)}^{2}d\tau , \end{array}$$

where

(32) $$\begin{array}{c} \beta_{3}\neq 0,\\ \phi_{0}=I\frac{-2\omega \left(4\omega^{2}+a_{4}^{2}+2a_{4}\beta_{4}+4\beta_{3}^{2}\right)+i\left[-a_{4}^{3}+4\left(\beta_{3}^{2}-\omega^{2}\right)a_{4}+\left(-a_{4}^{2}+4\omega^{2}+4\beta_{3}^{2}\right)\beta_{4}\right]}{\left[4\omega^{2}+{\left(a_{4}-2\beta_{3}\right)}^{2}\right]\left[4\omega^{2}+{\left(a_{4}+2\beta_{3}\right)}^{2}\right]},\\ \beta_{3}=0,\\ \phi_{0}=I\frac{-2\omega \left(4\omega^{2}+8a_{4}\beta_{4}+a_{4}^{2}\right)+i\left[4\left(4\omega^{2}-a_{4}^{2}\right)\beta_{4}-a_{4}\left(4\omega^{2}-a_{4}^{2}\right)\right]}{{\left(4\omega +a_{4}^{2}\right)}^{2}}. \end{array}$$

${\bar{\phi }}_{0}$ is the complex conjugate of *ϕ*_0_, and *Φ*3 is defined in Equation (22).

According to the Adomian decomposition method [22], we let

(33) $$\begin{array}{c} e_{3}=\overset{\infty }{\underset{n=0}{\sum }}y_{n}, \end{array}$$

(34) $$\begin{array}{c} F_{2}=-e_{3}^{3}+\left(a+1\right)e_{3}^{2}=\overset{\infty }{\underset{n=0}{\sum }}A_{n}, \end{array}$$

where

(35) $$\begin{array}{c} A_{n}=\frac{1}{n!}\frac{d^{n}}{d\lambda^{n}}{\left[F_{2}\left(\overset{\infty }{\underset{n=0}{\sum }}\lambda^{n}y_{n}\right)\right]}_{\lambda =0},n=0,1,2,\cdots , \end{array}$$

are Adomian polynomials of order *n*, in terms of *y*_0_, *y*_1_, ⋯, *y*_*n*_, ⋯. The first several Adomian polynomials are given as

(36) $$\begin{array}{c} A_{0}=\left(a+1\right)y_{0}^{2}-y_{0}^{3},\\ A_{1}=2\left(a+1\right)y_{0}y_{1}-3y_{0}^{2}y_{1},\\ A_{2}=y_{0}\left[2\left(a+1\right)y_{2}-3y_{1}^{2}\right]-3y_{0}^{2}y_{2}+\left(a+1\right)y_{1}^{2},\cdots ,\cdots , \end{array}$$

Substituting Equations (33) and (34) into Equation (31) yields

(37) $$\begin{array}{c} \overset{\infty }{\underset{n=0}{\sum }}y_{n}=\phi_{0}e^{i\omega t}+{\bar{\phi }}_{0}e^{-i\omega t}+\int_{0}^{t}\Phi_{3}\left(t-\tau \right)\left(\overset{\infty }{\underset{n=0}{\sum }}A_{n}\right)d\tau . \end{array}$$

Then, the solution structure of Equation (37) can be written as

(38) $$\begin{array}{c} \begin{array}{c} y_{0}=\phi_{0}e^{i\omega t}+{\bar{\phi }}_{0}e^{-i\omega t},\\ y_{1}=\int_{0}^{t}\Phi_{3}\left(t-\tau \right)\left(\left(a+1\right)y_{0}^{2}-y_{0}^{3}\right)d\tau ,\\ y_{2}=\int_{0}^{t}\Phi_{3}\left(t-\tau \right)\left(2\left(a+1\right)y_{0}y_{1}-3y_{0}^{2}y_{1}\right)d\tau ,\\ \cdots ,\cdots ,\cdots . \end{array} \end{array}$$

In the calculations of *y*_1,2_, the terms including *e*^−*α*4^*t* can be discarded to simplify the expressions. For *β*_3_ ≠ 0, *y*_1,2_ is easily given as

(39) $$\begin{array}{c} y_{1}=w_{0}+\overset{3}{\underset{n=1}{\sum }}\left(w_{n}e^{in\omega }+\bar{w_{n}}e^{-in\omega }\right),w_{n}=f_{1}\left(h_{n},n\omega \right),n=0,1,2,3,\\ y_{2}=f_{1}\left(q_{0},0\right)+\overset{5}{\underset{n=1}{\sum }}\left(f_{1}\left(q_{n},n\omega \right)e^{in\omega }+\bar{f_{1}\left(q_{n},n\omega \right)}e{-}^{-in\omega }\right), \end{array}$$

where

(40) $$\begin{array}{c} f_{1}\left(x,y\right)=\frac{2x\left(i2y+\beta_{4}+a_{4}\right)}{\left[i\left(2\beta_{3}-a_{4}\right)+2y\right]\left[i\left(2\beta_{3}+a_{4}\right)-2y\right]},\\ h_{0}=2\left(a+1\right){\left|\phi_{0}\right|}^{2},\\ h_{1}=-3{\left|\phi_{0}\right|}^{2}\phi_{0},\\ h_{2}=\left(a+1\right)\phi_{0}^{2},\\ h_{3}=-\phi_{0}^{3},\\ q_{0}=2\left(a+1\right)\left({\bar{\phi }}_{0}w_{1}+\bar{w_{1}}\phi_{0}\right)-3\bar{\phi_{0}^{2}}w_{2}-6w_{0}{\left|\phi_{0}\right|}^{2}-3\bar{w_{2}}\phi 0^{2},\\ q_{1}=2\left(a+1\right)\left({\bar{\phi }}_{0}w_{2}+\phi_{0}w_{0}\right)-6w_{1}{\left|\phi_{0}\right|}^{2}-3\bar{\phi_{0}^{2}}w_{3}-3\bar{w_{1}}{\phi_{0}}^{2},\\ q_{2}=2\left(a+1\right)\left({\bar{\phi }}_{0}w_{3}+\phi_{0}w_{1}\right)-6w_{2}{\left|\phi_{0}\right|}^{2}-3\bar{\phi_{0}^{2}w_{0}},\\ q_{3}=2\left(a+1\right)\phi_{0}w_{2}-6w_{3}{\left|\phi_{0}\right|}^{2}-3\bar{\phi_{0}^{2}w_{1}},\\ q_{4}=2\left(a+1\right)\phi_{0}w_{3}-3\phi_{0}^{2}w_{2},\\ q_{5}=-3\phi_{0}^{2}w_{3}. \end{array}$$

Then, the approximate solution of Equation (31) can be expressed as

(41) $$\begin{array}{c} e_{3}=y_{0}+y_{1}+y_{2}. \end{array}$$

The approximate solution for the case of *β*_3_ = 0 can be derived in a similar way, which is not presented here. The validity of the above analysis results will be demonstrated in Section 4.

## 4. Numerical Simulations

**Figure 1:**
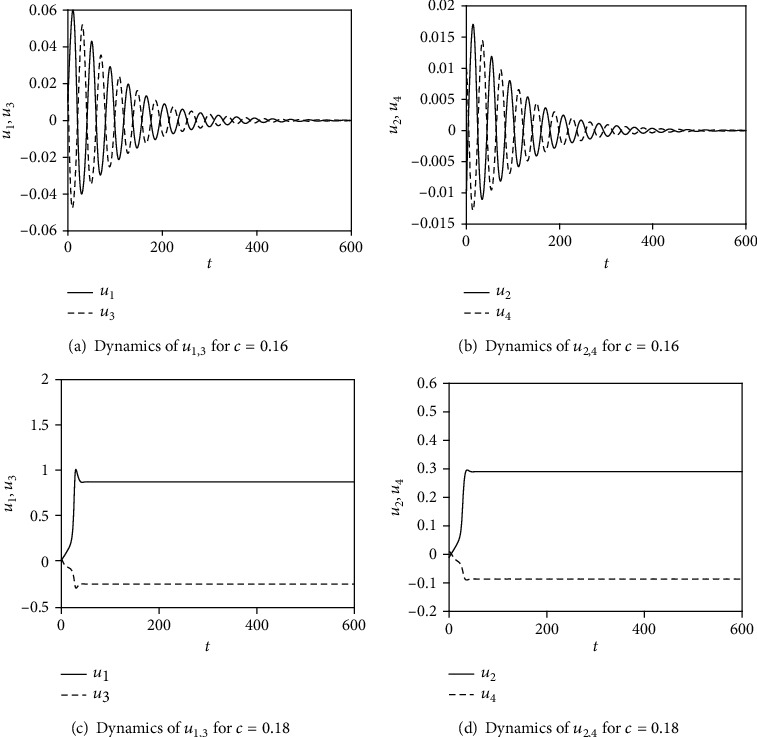
The dynamics of *u*_1,2,3,4_ in Equation (3) with *I* = 0. (a) The dynamics of *u*_1,3_ with *c* = 0.16. (b) The dynamics of *u*_2,4_ with *c* = 0.16. (c) The dynamics of *u*_1,3_ with *c* = 0.18. (d) The dynamics of *u*_2,4_ with *c* = 0.18. The initial conditions are taken as *u*_1_(0) = 0.01, *u*_2_(0) = −0.01, *u*_3_(0) = 0.02, and *u*_4_(0) = 0.01.

In this section, some numerical simulations for Equation (3) with *a* = 0.1, *b* = 0.08, and *γ* = 3 are carried out to demonstrate the effectiveness of the analysis in the last section. For the case of *I* = 0, the synchronization condition is *c* < (*a* + *bγ*)/2 = (0.1 + 3 × 0.08)/2 = 0.17 according to the analysis results in Equation (28). To illustrate the validity of the critical value of the coupling strength, *c* = 0.16 and *c* = 0.18 are chosen to carry out the numerical simulations for Equation (3) with *I* = 0. The initial conditions are taken as *u*_1_(0) = 0.01, *u*_2_(0) = −0.01, *u*_3_(0) = 0.02, and *u*_4_(0) = 0.01. The results are presented in Figure 1, which show that synchronous spikes do not occur for any given coupling strength in two coupled FHN neurons without external current.

**Figure 2:**
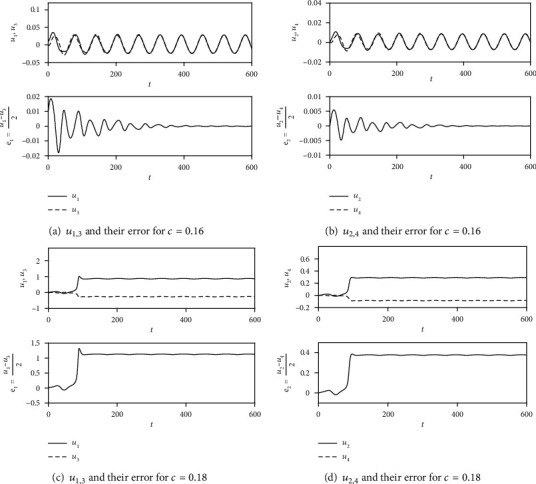
The dynamics of *u*_1,2,3,4_ and synchronization errors in Equation (3) with *I* = 0.01, *ω* = 0.1. (a) The dynamics of *u*_1,3_ and the error between them for *c* = 0.16. (b) The dynamics of *u*_2,4_ and the error between them for *c* = 0.16. (c) The dynamics of *u*_1,3_ and the error between them for *c* = 0.18. (d) The dynamics of *u*_2,4_ and the error between them for *c* = 0.18. The initial conditions are taken as *u*_1_(0) = 0.01, *u*_2_(0) = *u*_3_(0) = *u*_4_(0) = 0.

For the case of *I* ≠ 0, the current and frequency are chosen as *I* = 0.01 and *ω* = 0.1, respectively. The values of *a*, *b*, and *γ* remain the same. The initial conditions are taken as *u*_1_(0) = 0.01, *u*_2_(0) = *u*_3_ (0) = *u*_4_(0) = 0. The dynamics of *u*_1,2,3,4_ and synchronization errors *u*_1_ − *u*_3_, *u*_2_ − *u*_4_ are depicted in Figure 2 for *c* = 0.16 and *c* = 0.18, which demonstrate the effectiveness of the synchronization criterion *c* < (*a* + *bγ*)/2.

Next, we verify the effectiveness of analytical results of Equations (38)–(41) describing the synchronous dynamics of the two FHN neurons derived by using the Adomian decomposition method. The parameters in Equation (3) are given by *a* = 0.1, *b* = 0.08, *γ* = 3, *I* = 0.01, *ω* = 0.1, and *c* = 0.1, according to Equations (38)–(41); the approximation of *e*_3_ is expressed as

(42) $$\begin{array}{c} e_{3}=\left[\left(-0.0000891\;\cos\left(0.1t\right)+0.0003954\right)\cos\left(0.274t\right)+\left(-0.0001033\;\cos\left(0.1t\right)+0.00003\;\sin\left(0.1t\right)+0.003753\right)\sin\left(0.274t\right)\right]e^{-0.17t}+0.000853+0.001248\;\cos\left(0.1t\right)-0.001233\;\cos\left(0.2t\right)+0.00005934\;\cos\left(0.3t\right)+0.02617\;\sin\left(0.1t\right)-0.00003028\;\sin\left(0.2t\right)-0.0001005\;\sin\left(0.3t\right). \end{array}$$

**Figure 3:**
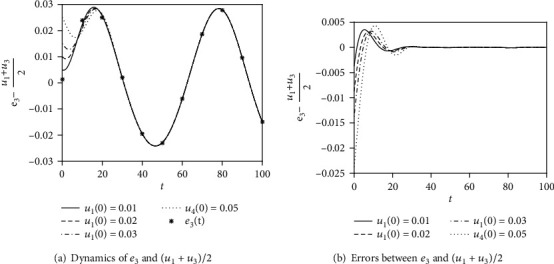
The comparison between numerical and analytical results of synchronous dynamics *e*_3_ = (*u*_1_ + *u*_3_)/2 in Equation (3) with *I* = 0.01, *ω* = 0.1 for different initial conditions. (a) The synchronization dynamics of *e*_3_ and (*u*_1_ + *u*_3_)/2. (b) The errors between *e*_3_ and (*u*_1_ + *u*_3_)/2. The initial conditions are *u*_2_(0) = *u*_3_(0) = *u*_4_(0) = 0, while *u*_1_(0) varies.

The comparison between the numerical results of Equation (3) using the Runge-Kutta method for different initial conditions and the analytical results from Equation (42) is given in Figure 3. Clearly, the analytical results coincide exactly with the numerical ones after enough time, which show the correctness of the analytical results expressed in Equation (42).

## 5. Conclusions

In this paper, the synchronization of two FHN neurons with or without external current is investigated by using the Laplace transform and the Adomian decomposition method. A linear transformation is carried out for the original system to obtain the synchronization error system. Different from other methods, the error system is converted into sets of Volterra integral equations by using the convolution theorem. Then, the successive approximation method in the integral equation theorem is used to judge whether the synchronization error approaches to zero. Thus, a criterion is obtained to determine the occurrence of synchronization in the two FHN neurons.

It is found that the two coupled FHN neurons without external current can only maintain neural rest when synchronization is achieved. While the external current is not zero, synchronous spikes occur. The numerical simulations demonstrate that the criterion presented in this paper is very effective. The analytical result describing the synchronous dynamics of the two FHN neurons derived by using the Adomian decomposition method is accurate enough. The analytical expression helps us to deeply understand the synchronous dynamics of coupled FHN neurons. The method adopted in this paper is valid to discuss the synchronization between FHN neurons. Moreover, the calculation quantities of this method are small, which can be developed to judge synchronization between *N* coupled FHN neurons.

## Data Availability

All data generated or analyzed during this study are included in this article.
